# From Spectra to Signatures: Detecting Fentanyl in Human Nails with ATR–FTIR and Machine Learning

**DOI:** 10.3390/s25010227

**Published:** 2025-01-03

**Authors:** Aubrey Barney, Václav Trojan, Radovan Hrib, Ashley Newland, Jan Halámek, Lenka Halámková

**Affiliations:** 1 Department of Environmental Toxicology, Texas Tech University, Lubbock, TX 79409, USA; aubbarne@ttu.edu (A.B.); asnewlan@ttu.edu (A.N.); jan.halamek@ttu.edu (J.H.); 2 Cannabis Facility, International Clinical Research Centre, St. Anne’s University Hospital Brno, 60200 Brno, Czech Republic; vaclav.trojan@fnusa.cz (V.T.); radovan.hrib@fnusa.cz (R.H.); 3 Department of Natural Drugs, Faculty of Pharmacy, Masaryk University, 60200 Brno, Czech Republic; 4 Center for Pain Management, Department of Anesthesiology and Intensive Care, St. Anne’s University Hospital Brno, 60200 Brno, Czech Republic

**Keywords:** fentanyl, fingernails, toenails, ATR–FTIR, machine learning, PLS-DA, SVM-DA

## Abstract

Human nails have recently become a sample of interest for toxicological purposes. Multiple studies have proven the ability to detect various analytes within the keratin matrix of the nail. The analyte of interest in this study is fentanyl, a highly dangerous and abused drug in recent decades. In this proof-of-concept study, ATR–FTIR was combined with machine learning methods, which are effective in detecting and differentiating fentanyl in samples, to explore whether nail samples are distinguishable from individuals who have used fentanyl and those who have not. PLS-DA and SVM-DA prediction models were created for this study and had an overall accuracy rate of 84.8% and 81.4%, respectively. Notably, when classification was considered at the donor level—i.e., determining whether the donor of the nail sample was using fentanyl—all donors were correctly classified. These results show that ATR–FTIR spectroscopy in combination with machine learning can effectively differentiate donors who have used fentanyl and those who have not and that human nails are a viable sample matrix for toxicology.

## 1. Introduction

Human nails have recently come forward in the world of toxicology as an alternative matrix to hair and bodily fluids [1,2,3,4,5,6]. These matrices can maintain foreign substances and respective metabolites within their structure that may indicate prior illicit substance use or abuse. When substances such as alcohol or illicit drugs enter the body, they are absorbed into the bloodstream, distributed, and deposited throughout the body, with the potential for biotransformation. Blood flow to the nail bed leads to the deposition of these substances in the nails as they grow, making nails a valuable resource for detecting past substance use [4]. Although the keratinized structure of the nail is mainly used for nail bed protection, the pores found within the structure trap substances within the nail matrix that cannot be easily removed by daily hygienic routines [1,2,3,5,7]. These substances can remain within the keratin matrix for approximately 3–8 months, but some may remain for up to a year [1,2,3,5,7]. When compared to other matrices, the period that substances remain in the keratin structure is just one of the many advantages that nails have. Other advantages include noninvasive collection and storage procedures [7,8]. Nails grow continuously and are not affected by growth cycles or color bias via melanin, unlike hair [5]. This is what makes nail sample collection, storage, and preparation efficient, as there is very little that will impact the collection or analysis procedures. Additionally, the deposition of substances into the nail can alter the chemical composition, but it does not disrupt the overall integrity of the keratin matrix, providing distinguishability between the natural composition of the nail and the target analyte [8,9]. These advantages are what recently caused an interest in researching nails as a matrix for toxicological tests. The results of this type of research will make nails a useful and appealing tool for toxicology, forensic science, and medical practices.

There are two main methods by which a foreign substance is incorporated into the nail [4]. As mentioned before, substances may be absorbed into the bloodstream and internally deposited into the nail through blood vessels in the nail bed [4]. Alternatively, nails can be externally contaminated through sweat or direct contact with a substance [4]. Although some research has been conducted on external deposition, such as with explosive residue, previous research has generally focused on internal deposition through the use of gas or liquid chromatography with mass spectrometry to analyze nails for alcohol, drugs, and other substances in subjects of all ages [10,11,12]. Kintz, Gheddar, and Raul tested for and quantified multiple anabolic steroids in human nails [7]. Engelhart and Jenkins published a study in which they tested postmortem nail clippings of suspected drug users for opiates [2]. Shu et al. detected numerous drugs in nail samples over three years [5]. In addition to these studies, nails have also been used as a sample matrix for substances such as nicotine and sedatives [10,12,13]. Although gas and liquid chromatography have been the main avenues for nail analysis, spectroscopic methods, such as FTIR and Raman spectroscopy, have proven to be appealing due to non-rigorous sample preparation, ease of use, and non-destructive nature [6]. FTIR and Raman spectroscopy have been utilized to analyze and distinguish human nail clippings containing substances such as chlorine and alcohol. These methods have also been applied to predict biological information, such as the sex of the donor [6,9,14].

The analyte of interest in this study is fentanyl, a synthetic opioid [15]. In recent decades, there has been an alarming rise in overdose cases involving fentanyl and its analogues or fentanyl-related substances (FRS) [15,16,17]. First created in 1960 as a potent analgesic drug, fentanyl is now illicitly manufactured, distributed, and combined with or sold as other illicit substances [15,18]. In more recent years, fentanyl analogues, which have varying potency, emerged as alternatives to the original substance [16]. The potency and toxicity of fentanyl and FRS present a danger to not only drug users but also drug manufacturers, drug dealers, first responders, medical personnel, law enforcement, and the public.

The increasing prominence of these substances and their fatal effects on society have made them a prominent target for research, primarily focusing on detection methods. Current detection methods for fentanyl typically include gas or liquid chromatography–mass spectrometry (GC/LC–MS), colorimetric analysis, or lateral flow assays [15,19]. The most common detection method for research and casework is LC–MS, as urine and blood are the typical biological samples utilized for toxicological purposes. Rab, Flanagan, and Hudson published a study on fentanyl and FRS detection using liquid chromatography and high-resolution mass spectrometry in which they focused on post-mortem cases [15]. As with nail analysis, the main issue with current methodologies is that it may require extensive sample preparation. Attenuated total reflectance–Fourier transform infrared (ATR–FTIR) spectroscopy has the advantages of being nondestructive, noninvasive, and a relatively simplistic instrumental procedure, and it requires very little, if any, sample preparation. FTIR consists of measuring the amount of infrared light absorbed by a sample. The resulting spectra provide valuable insight into the molecular structure of the sample being analyzed. Various peaks that may appear on the spectrum can be assigned to specific chemical functional groups. The spectra obtained from FTIR may be analyzed using any number of machine learning techniques [14,20,21,22].

Additionally, FTIR has been applied practically. Particularly, several sources have discussed in detail how FTIR can be a rapid form of drug screening. Specifically, numerous sources discuss fentanyl detection within a mixture of substances [23,24,25,26]. Ramsay et al. combined a portable ATR–FTIR spectrometer (Agilent 4500a, Agilent Technologies, Santa Clara, CA, USA) with PLS methodology to build a model to predict fentanyl content within drug mixtures [25]. While this research focuses solely on fentanyl detection rather than quantification, this methodology can be applied to samples such as those implemented in this study. Prior research has evaluated the efficacy of FTIR spectroscopy to detect fentanyl within samples and found high specificity rates of around 90% [23,26]. This shows that FTIR is not only capable of detecting fentanyl within samples but also is ideal for differentiating samples that do not contain fentanyl.

The purpose of this research was to utilize ATR–FTIR spectroscopy together with PLS-DA and SVM-DA machine learning techniques to determine if nail clippings containing fentanyl can be differentiated from those without. This study serves as a proof-of-concept, building upon previous work in which an identical methodology was applied with the target analyte of alcohol and, in another study, to predict sex from fingernail samples with a high degree of accuracy [9,14]. In this paper, it is shown that ATR–FTIR used with machine learning can differentiate and correctly classify control and fentanyl-containing nail samples with a high degree of sensitivity, specificity, and overall accuracy. By combining ATR–FTIR with PLS-DA and SVM-DA, we successfully identified complex patterns within the spectral data. This enables robust and reliable detection of substances, even in complex matrices, making it a powerful tool for analysis.

## 2. Materials and Methods

Sample Collection and Preparation: Nail clippings were collected from 79 volunteers of various ages, sex, and ethnicities (63 regular donors and 16 fentanyl donors). The eligibility criteria for volunteer donors included healthy nails with no signs of skin or nail disease. Nail samples containing fentanyl were collected from patients at the Centre for Pain Management, Department of Anesthesiology and Intensive Care at St. Anne’s University Hospital in Brno, Czech Republic. All selected donors from this location were on medication for fentanyl citrate for an extended period and had their medical histories reviewed prior to sample collection. The literature and established testing protocols for various analytical laboratory techniques confirm that fentanyl deposits in nails when ingested, regardless of whether the source is illicit or prescribed medication [27]. Samples consisted of 1–5 nail clippings from each donor. For our study, we collected nail clippings from different fingers of each participant to ensure a comprehensive representation of potential substance accumulation in the nails. The samples were stored in a plastic Ziploc bag along with a label containing relevant information, such as patient ID number, sample collection date, biographical information, history of drug and alcohol use, chronic diseases, and medications. Each nail clipping was rinsed with ≥99.5% acetone prior to collecting spectra to remove any exterior surface contaminants. This study and sample collection were approved by the Institutional Review Board of Texas Tech University under No. IRB2022-211.

ATR–FTIR Spectroscopy: Spectra were collected using a Nicolet iS20 (Thermo Scientific, Waltham, MA, USA) spectrometer equipped with a Smart iTX diamond ATR accessory in tandem with OMNIC software (version 9.12.1019). A total of 15 spectra of 32 scans per spectra were collected for each sample. Each spectrum was taken in a range of 400–4000 cm^−1^ with a resolution of 4. We have excluded spectral regions between 1801 cm^−1^ and 2599 cm^−1^ from our analysis to reduce any potential interference caused by the diamond attenuated total reflection (ATR) crystal. To account for the heterogeneous nature of the keratin matrix, each spectrum was taken from a different area of the nail clipping. By sampling spectra from the left, middle, and right regions of the nail on both the dorsal (top) and ventral (underside) surfaces, we ensured that the potential variability in composition and structure within the nail was adequately represented in our analysis. The diamond accessory was cleaned with ≥99.5% acetone and allowed to dry between samples, and a background spectrum was taken prior to new sample spectral analyses. Force was applied to each sample to ensure sufficient contact with the diamond accessory. ATR–FTIR spectroscopy integrates with machine learning algorithms by treating each wavenumber in the spectral data as a unique feature, resulting in a dataset where the number of features matches the number of wavenumbers measured. Prior to the modeling, the spectral data underwent several preprocessing steps to improve interpretability. These steps involved transforming transmission values to absorbance using the formula log(1/T), applying a second-order derivative transformation with a second polynomial, normalizing by the total area, and performing mean centering. The modeling was conducted using MathWorks MATLAB R2024b (version 24.2.0.2712019, Natick, MA, USA) in conjunction with the PLS Toolbox 9.5 from Eigenvectors Research Inc. (Manson, WA, USA).

PLS-DA: Partial least squares (PLS) is a versatile linear regression method that can be effectively adapted for classification tasks. When used with a dummy response variable, the technique becomes known as partial least squares discriminant analysis (PLS-DA) [28]. In PLS-DA, the primary difference is the addition of a thresholding step to the predicted y-values to assign class labels, such as distinguishing between “Fentanyl” and “Control” samples. The PLS algorithm reduces the data’s dimensionality by transforming the original predictors, such as a full FTIR spectrum, into a smaller set of latent variables (LVs) or components. These LVs are selected to maximize the covariance between the response variable (class assignment) and new linear combinations of the original predictors while minimizing the influence of irrelevant variables [22]. An important step in PLS-DA is determining the optimal number of LVs, ensuring that the model captures only the variability relevant to predictions without incorporating noise [29]. This is typically achieved by constructing several PLS models with different numbers of LVs and using cross-validation to identify the model that minimizes classification error. The number of LVs that resulted in the lowest classification error rate during cross-validation was chosen for the final PLS-DA model [14].

PLS-DA effectively handles high-dimensional datasets and provides a robust classification performance based on linear relationships between the predictors and the response variable. Due to the complexity of biological data, it was hypothesized that SVM, a non-linear approach to data prediction, could optimally capture patterns potentially left unrecognized by PLS linear predictions. To ensure robust performance for both the PLS-DA and SVM-DA models, the dataset was split into two subsets: a training set (975 spectra) and a test set (210 spectra), with the latter held strictly for external validation. The training dataset was used to tune hyperparameters through a cross-validation process, specifically employing a ten-fold Venetian blind scheme, optimizing the model’s performance before testing it on the held-out test set. This method involves splitting the training data into ten equally sized subsets or “folds”. During each iteration of the cross-validation process, one of these ten subsets was temporarily set aside as the validation set, while the remaining nine subsets were used to build a preliminary model.

This model was then used to predict the class labels for the excluded validation set, such as “Fentanyl” or “Control”, and the classification error rate was recorded. This process was repeated ten times, each time excluding a different subset as the validation set until all subsets had been used once for validation. Then, the average PLS-DA classification error was calculated. This process was repeated across various models with an increasing number of LVs to identify the optimal number that minimized the classification error. After determining the ideal number of LVs for the PLS-DA model, the specific number of LVs was applied to build the final model using the entire training dataset. This final model was subsequently applied to the test set, which was kept separate from the model-building and cross-validation stages to assess the predictive accuracy of unseen data.

Identical steps were followed for the SVM-DA model-building process, using training, test splitting, and Venetian blind cross-validation procedures to fine-tune the model’s hyperparameters (cost and gamma hyperparameters). This approach ensured that the PLS-DA and SVM-DA models were rigorously validated and allowed for a fair comparison of their performance in terms of predictive accuracy and generalizability to new, unseen data.

SVM-DA: It has been shown that machine learning methods, particularly those capable of modeling nonlinear relationships between variables, such as support vector machine (SVM), can provide higher classification accuracy. In our study, we first used PLS-DA, a standard technique for linear classification, to model the relationship between spectral data and class labels. While effective for high-dimensional datasets, PLS-DA assumes linear relationships, which may not fully capture the complexity of biological data. Hence, we extended our approach further and implemented the support vector machine discriminant analysis (SVM-DA) model, capable of modeling nonlinear relationships, to observe whether this will improve the accuracy of classification. Accordingly, SVM-DA was run to give more emphasis to the non-linear interactions in the data that may not have been captured by the linear approach of PLS-DA. The SVM-DA model input features were the scores from the LVs obtained from the PLS-DA model to allow for the dimensionality reduction provided by PLS-DA while using the non-linear classification capabilities of SVM. The LVs effectively captured variance in this data, serving as the features.

As a supervised learning technique, especially suitable for classification purposes, SVM-DA can easily classify two classes in diagnostic studies. First proposed by Vapnik in 1995, the SVM-DA technique has been extensively applied to many scientific fields, such as bioinformatics, with successful solutions to various diagnostic problems [30,31,32,33]. The SVM-DA methodology searches for an optimum set of support vectors that define a hyperplane in the feature space. These vectors are identified during the training process, and the model then fits the data using a kernel function of choice selected by the user. The kernel modifies the behavior of the SVM-DA model to optimize the hyperplane’s position, ensuring maximum separation (or margin) between the classes. In this work, an RBF kernel was implemented and optimized through a Venetian blind cross-validation process combined with a systematic grid search over the model hyperparameters (cost and gamma hyperparameters).

A regular grid search was conducted to find the best parameters for the model by using Venetian blind cross-validation with ten splits. This method assisted in model performance evaluation across different subsets of the data and generalization of the model because the systematic grid search is performed over different parameter combinations across subsets. The cost parameter controls the trade-off between the low error on the training data and model simplicity to prevent possible overfitting or underfitting. Similarly, the gamma parameter, which defines the range of influence for a single training example, was optimized in such a way that the model would be able to learn from general underlying patterns within the data without being too sensitive to noise [34].

Model Evaluation: In our analysis, sensitivity, specificity, and overall accuracy were calculated at all stages of model building, including calibration, cross-validation, and external validation. An independent test dataset, set aside at the beginning of the statistical analysis, was used for external validation to determine the performance of both the PLS-DA and SVM-DA models. This enabled an independent assessment of model generalization. Further, receiver operating characteristic (ROC) curves were plotted to compare the results of cross-validation and external validation for both approaches to provide the discriminative capabilities for both models. The ROC analysis presents the possibility of exploring the trade-offs between sensitivity and specificity through various threshold settings, providing information about the area under the curve (AUC) for each model. The AUC measures how well a model can distinguish between classes. An AUC of 0.5 suggests the model performs no better than random guessing, while a model with an AUC close to 1.0 gives a high performance in classification. It evaluates the ability of the model to separate the classes at different threshold values. By comparing these results from cross-validation and external validation, the generalizability was assessed for both the PLS-DA and SVM-DA models. While cross-validation estimates the performance on subsets of the training data, with the main objective of model parameter tuning, external validation tests the model on completely unseen data to provide a more realistic estimate of predictive accuracy. This helps ensure the model is not overfitting and will perform well on independent datasets. Ideally, the results obtained from cross-validation and external validation will not be notably different, since large differences could reflect overfitting or poor generalization. The consistency of these results will provide evidence of a robust model.

## 3. Results

**Figure 1:**
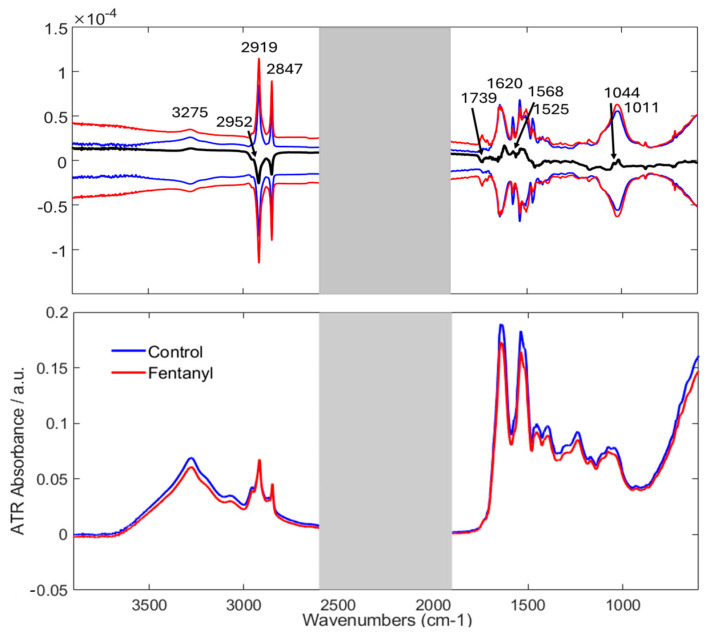
Top: Difference spectrum of “Control” and “Fentanyl” mean spectra, highlighting small differences between the groups. The standard deviation (SD) spectra for each group are also shown to highlight the variability in the spectral data. The SD spectra for the “Control” group (shown as a blue line) and the “Fentanyl” group (shown as a red line) are plotted in both positive and negative directions around the difference spectra. This visual representation offers a clearer understanding of the range of spectral variability in each group and helps to clarify the extent of the differences observed between the two classes. Bottom: Raw mean spectra of “Control” and “Fentanyl” groups.

Visual Examination of Spectra: The bottom graph of Figure 1 shows the mean ATR–FTIR spectra of fingernails from the two groups of volunteers. It is observed that these spectra exhibit peaks at similar positions with only minor differences in their intensities. Since the basic composition of human nails does not vary, slight differences due to fentanyl consumption are noticeable between the groups. However, the average spectra show very small differences, and the high standard deviation is larger than the differences indicated by the difference spectra (Figure 1, top). Because of this, simply relying on visual comparisons would not be very effective. Therefore, advanced multivariate statistical techniques were employed for more reliable differentiation. The spectral profile of human nails shows characteristic vibrational frequencies corresponding to various biomolecular components, thus offering valuable insights into the characteristics of human nails. Major regions within the nail spectra are dominated by signals corresponding to nucleic acids in the region of 1000–1250 cm^−1^, lipids at 2800–3000 cm^−1^, and carbohydrates at 800–1000 cm^−1^. The detailed peak assignments for molecular vibrations in human nails are reported in the literature and, most specifically, in our previous study [14].

Although the spectral profiles show some similarities, the difference spectrum (Figure 1, top) obtained by comparing the mean spectra of the “Fentanyl” and “Control” groups highlights prominent peaks. These variations could be partially attributed to the ATR–FTIR spectral bands identified in our earlier research associated with changes in Amide I (1620 cm^−1^) and Amide II (1525 cm^−1^) [20]. The Amide I band is generally one of the most prominent features in protein spectra, with variations in this region often linked to changes in the protein’s backbone structure [35]. A significant difference also noted occurred in the Amide II band, which is a highly complex region where identifying structural changes can be challenging. Significant variations in intensity were also noted in the band identified in the spectrum at 1044 cm^−1^ assigned to S–O bonds of the cysteic acid unit [36].

Compared to the FTIR spectra measured in our previous studies, the difference spectrum exhibited distinct peaks, particularly at wavenumbers 2952 cm^−1^, 2919 cm^−1^, and 2848 cm^−1^. These peaks were identified in our earlier studies as well but are also characteristic of fentanyl spectra as described in the literature [37]. Notably, these peaks correspond to lipids in human nail composition but overlap with those identified in fentanyl [21]. Thus, fentanyl can only introduce subtle changes in the FTIR spectrum. These changes might be masked or overlapped by the inherent signals of lipids in the nail matrix. The overlapping spectral features emphasize the need for advanced analytical methods, such as machine learning algorithms, which can be trained on labeled samples to identify specific patterns associated with fentanyl. ML algorithms can enhance the detection of subtle differences, even in the presence of complex matrices. This approach not only improves classification accuracy but also provides a more robust framework for interpreting the spectral data, thus aiding in the differentiation of fentanyl from non-specific changes related to nail matrix components.

PLS-DA Classification: To identify fentanyl donors based on spectral signatures rather than individual peak intensities, the PLS-DA method was adopted to develop a robust model. This method searches for regular patterns across labeled spectra to enable the system to learn features and make predictions on unknown data. The “Control” class contained 63 donors, while the “Fentanyl” class had 16 donors. The total amount of labeled spectra used for the training in the PLS-DA model was 975. In PLS-DA, there are two important ways to ensure the model performance is sufficient: the selection of the number of optimal LVs and the setting of the classification threshold. The optimal number of LVs was determined using the method of cross-validation with 10-fold Venetian blind cross-validation. Eight LVs were selected for further analysis.

The calibration set resulted in a sensitivity of 93.9%, specificity of 91.9%, and an overall accuracy of 92.3%. The classification threshold was developed based on the distribution of calibration-sample predictions; spectra that fell above that classification threshold were predicted as “Control”, and those falling below the threshold were predicted as “Fentanyl”. The threshold is optimized for metrics such as sensitivity and specificity under the assumption that the predicted values for each class are approximately normally distributed, ensuring that the model generalizes effectively on new data. During cross-validation, the results turned out slightly lower: sensitivity of 88.9%, specificity of 89.3%, and accuracy of 89.2%. However, these results reflected a very robust performance by the model and showed high sensitivity and specificity to discriminate between the two donor groups, showing its potential applicability in the identification of fentanyl use through spectral analysis.

**Figure 2:**
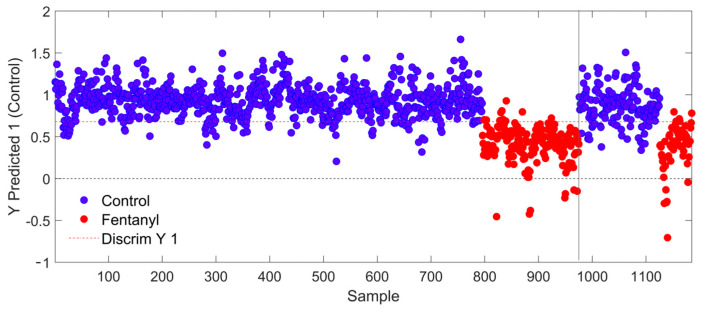
PLS-DA model results for all training and test spectra using 8 LVs. All spectra above the discrimination threshold are predicted as “Control”, while those below the threshold are predicted as “Fentanyl”. Blue data points represent spectra from samples which contained no fentanyl, while red data points represent spectra from samples which were known to contain fentanyl.

After training the PLS-DA model with eight LVs to differentiate between “Control” and “Fentanyl” nail specimens, the model predicted spectra from an independent test set that had not been introduced during model training. This approach provided an unbiased evaluation of the final PLS-DA model. In Figure 2, there are the discriminant scores for each nail spectrum predicted during calibration (the left side up to Sample 975) as well as during external validation (the right side). Figure 2 also displays the prediction scores for each spectrum alongside the classification threshold (indicated by the red dashed line). Spectra above this threshold are classified as “Control”, while those below the line are classified as “Fentanyl”. The external validation results showed a sensitivity of 82.6%, specificity of 91.7%, and overall accuracy of 84.8% based on spectral level, confirming the model’s robust performance on new data. The PLS-DA model achieved 100% accuracy in distinguishing between the two groups at the donor level using the standard 50% threshold, meaning every donor was correctly classified with no errors.

While the PLS-DA model benefits from being easily interpretable and linear, it is inherently limited to modeling linear relationships between variables [38]. Since biological data often display non-linear characteristics, more sophisticated non-linear machine learning techniques could potentially offer better performance in these cases. However, PLS-DA remains a robust method for linear discrimination, particularly when dimensionality reduction and model interpretability are key objectives.

SVM-DA Classification: The classification problem was solved by the support vector machine model, which used a radial basis function kernel, or RBF, with optimal parameters: cost equals 3.1623 and gamma equals 0.031623. The model was trained using 210 support vectors, which indicates its strength in the representation of the data for sufficient classification. The SVM-DA model effectively turns its raw output into probabilities, which then direct the classification decision for each spectrum. The SVM-DA model has calculated a probability for both classes (“Control” and “Fentanyl”) and picked the one with the higher probability as the final prediction of that spectrum. Once the predictions for each spectrum were obtained, true labels, sensitivity, specificity, and accuracy were evaluated to determine the performance of the SVM-DA classifier. The performances of the SVM-DA classification model for three stages were assessed: calibration, cross-validation, and external validation. Each step provides key information about the model’s ability to discriminate between control and fentanyl spectra. During model calibration, the sensitivity was 97.6%, and the specificity was 81.7%. The overall accuracy during calibration was 94.7%, demonstrating that the model effectively recognizes both classes.

**Figure 3:**
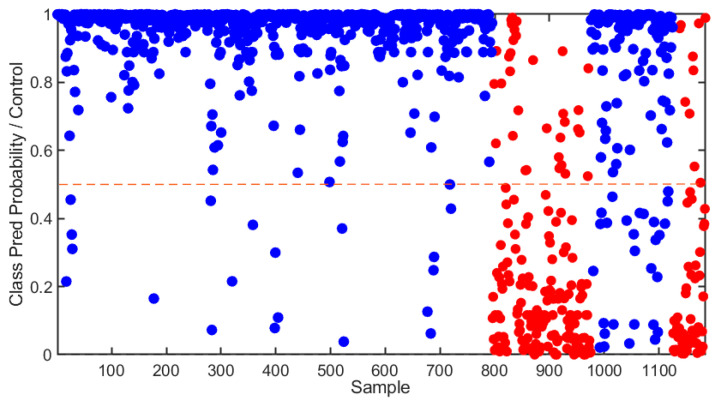
SVM-DA results of all training and test spectra. All spectra above the threshold are predicted as “Control”, while those below the threshold are predicted as “Fentanyl”. Blue data points represent spectra from samples which contained no fentanyl, while red data points represent spectra from samples which were known to contain fentanyl.

During cross-validation, the sensitivity for the control class decreased slightly to 94.7%, and the specificity decreased to 77.8%. The accuracy in this phase was 91.6%, indicating the model’s continued reliability when validated on various subsets of data. The model sensitivity during external validation was 81.3% for the control class, while the specificity was 81.7%. During this stage, the overall accuracy was 81.4%, hence showing a small drop in performance during calibration and cross-validation. This means that, while the model still predicts the most probable class for each spectrum fairly well, there are challenges in consistently achieving high accuracy. The results of the SVM-DA model are shown in Figure 3. At the donor level, the SVM-DA model achieved 100% accuracy in distinguishing between the two groups.

In general, these results reflect that the SVM-DA model has a robust classification capability regarding control and fentanyl spectra across all stages. High sensitivity and specificity, accuracy of calibration, cross-validation, and independently performed external validation confirm the model’s efficiency in distinguishing between the two classes effectively.

Receiver Operating Characteristic (ROC) Analysis: To further explore and compare the performance of the PLS-DA and SVM-DA models, ROC curve analysis was conducted. ROC curves are a standard technique for comparing the diagnostic performance of classification models where the sensitivity or true positive rate is plotted against specificity or true negative rate to evaluate different classification thresholds. The area under the curve (AUC) represents the overall accuracy of the model in distinguishing between classes. For the present study, AUCs were calculated using the trapezoidal method of integration; the 95% confidence interval (CI) was calculated using the method described by De Long et al. [39]. ROC curves were created using the cross-validation and external validation probability estimates.

**Figure 4:**
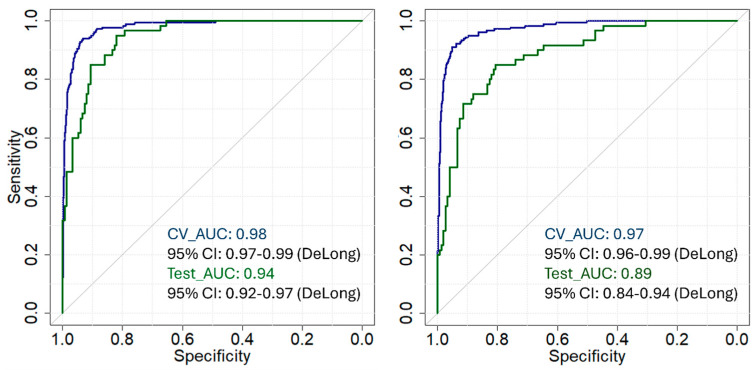
(**Left**) ROC analysis for PLS-DA showing overall accuracy values of 98% and 94% for the cross-validation and external validation, respectively. (**Right**) ROC analysis for SVM-DA showing overall accuracy of 97% and 89% for cross-validation and external validation, respectively.

The ROC curve for the PLS-DA model (Figure 4, left) has an AUC value of 0.98 with a 95% CI between 0.97 and 0.99, respective to cross-validation. This represents excellent discriminatory ability between Controls and Fentanyl cases in the training data. In the external validation set, the PLS-DA model has an AUC value of 0.94, with a 95% CI between 0.92 and 0.97, showing high performance on the test dataset but a slight decrease when compared to the cross-validation results.

On the contrary, the AUC for the SVM-DA model’s cross-validation (Figure 4, right) was 0.97, with a 95% CI between 0.96 and 0.99. This is slightly lower than the PLS-DA model’s AUC but still shows high performance. The SVM-DA model has an AUC of 0.89, with a 95% CI of 0.84 to 0.94 for the external validation set, which is lower compared to the PLS-DA model. The SVM-DA model, though still having excellent performance in the training data, shows reduced performance during external validation data compared to the PLS-DA.

In general, the PLS-DA model has better performance in external validation compared to the SVM-DA model, while the latter had an overall sufficient calibration performance. This may indicate the sensitivity of SVM-DA models to hyperparameters and possible overfitting that affects the generalization capability of new data.

## 4. Discussion

When using ML to analyze FTIR spectra of nails to identify fentanyl users, it is important to recognize that both fentanyl and its metabolite, norfentanyl, will contribute to the spectral data. In nails, it is possible to identify fentanyl and norfentanyl due to the direct deposition of the drug into the keratin matrix [27]. Distinguishing fentanyl from its metabolites within nails can be challenging because the metabolites might have similar or overlapping FTIR peaks as the parent compound. Exact identification often depends on the specific chemical changes occurring during metabolism and the resolution of the FTIR technique. Additionally, fentanyl use can cause broader biochemical changes in the overall structure of the nail, which may affect the intensity and pattern of peaks in the spectra, further supporting differentiation between the fentanyl group and the control. In practice, while peaks might overlap, advanced analytical methods or additional complementary techniques can help differentiate between fentanyl and its metabolites. However, distinguishing between fentanyl and its metabolites may be more relevant in specific forensic investigations, where knowing the exact drug exposure timeline or understanding the nature of a person’s drug use is important. Our focus is on identifying patterns of fentanyl use in general without differentiating between fentanyl and its metabolites.

In this study, PLS-DA and SVM-DA techniques were applied to FTIR spectra of nail samples and showed successful differentiation between samples containing fentanyl, metabolites, and other changes in the nail matrix caused by long-term use and those that did not contain the target analytes. The PLS-DA model was able to classify samples with an overall accuracy of approximately 84.8%, while the SVM-DA model was able to classify samples with an overall accuracy of approximately 81.4% during external validation. Although the PLS-DA model classified newly introduced samples more accurately, the SVM-DA model was better at assessing nonlinear relationships and had a higher calibration accuracy. This suggests that, while the PLS-DA model may perform better in external validation, the SVM-DA model’s stronger calibration may offer better sensitivity to underlying patterns in the training data, which could be important for complex datasets later in research. These models may be fine-tuned further to possibly achieve an even higher accuracy. Thus, the current results demonstrate that ATR–FTIR combined with machine learning may be used for noninvasive and rapid fentanyl screening to indicate prolonged use. This also supports human nails as a new ideal matrix for toxicological and forensic purposes. This research lays the foundation for new research into the development of novel methods for fentanyl detection utilizing nail clippings.

A possible limitation of our study is the exclusive use of fentanyl citrate in recruited donors for the fentanyl group, while numerous fentanyl analogues are also present in illicit drugs. FRS differ structurally from fentanyl citrate, potentially leading to variations in their ATR–FTIR spectra. As a result, the current ML models, trained solely on fentanyl citrate data, may generalize less effectively to detect other fentanyl derivatives. However, a previous study conducted by Crepeault et al. [40] suggested that the differences in FTIR spectra among various FRS are relatively minor. The authors used data collected between May 2018 and July 2021 at supervised consumption sites in Vancouver and Surrey, BC, where drug-checking services were provided. This study employed FTIR reference libraries to identify fentanyl and its analogues in the supplied samples. Although they were able to reliably detect fentanyl or FRS, differentiating between the analogues and fentanyl itself was impossible due to the spectral similarities between them. Future studies will focus on expanding the dataset to include these analogues, enabling the development of a more robust model that can identify a broader range of fentanyl derivatives commonly encountered in forensic contexts. Despite this limitation, our findings represent an important step toward noninvasive detection of fentanyl use, with future enhancements expected to broaden applicability.

One important consideration in this study is the potential confounding effects of other medications that some donors were taking during sample collection. Medications other than fentanyl could alter the biochemical composition of the nails, affecting the ATR–FTIR spectra and potentially interfering with the detection results of fentanyl and metabolites. Many drugs that are metabolized and excreted through the body’s various keratinized tissues (like nails) may be deposited within the keratin matrix, similar to fentanyl. These deposits could introduce changes in spectral peaks, mainly by alternating the intensity of existing peaks, potentially overlapping with those indicative of fentanyl use. In this study, we did not control for or systematically account for the presence of other medications in the donor population, so there is a risk that some of the differences detected between the control and fentanyl user groups could be influenced by these other substances. This confounding factor may reduce the specificity of our machine learning model, as the model may mistakably learn patterns from unrelated medications rather than from fentanyl-specific biochemical changes. However, the model’s performance during external validation, which was rigorously designed, supports the conclusion that the model primarily learned patterns specific to fentanyl use. The model showed excellent performance in distinguishing fentanyl users from the control group, suggesting that it successfully identified fentanyl-related biochemical changes in the nail matrix rather than results significantly influenced by unrelated medications. This strengthens the argument that the proposed approach is robust and capable of detecting fentanyl-specific signatures even in the presence of other substances. To exclude the possibility that the model could have been influenced by medications other than fentanyl, validation studies will be conducted.

## 5. Conclusions

In conclusion, this proof-of-concept study demonstrates the feasibility of using ATR–FTIR combined with machine learning techniques to detect fentanyl in human nails, forming a promising basis for noninvasive detection methods. These results, based solely on spectral data, show that, while the PLS-DA and SVM-DA models achieve external validation accuracies of 84.8% and 81.4%, respectively, the classification accuracy reaches 100% when analyzing at the donor level. This distinction highlights the potential of these methods to effectively differentiate between fentanyl users and control groups when considering individual donor information. Although the exclusive use of fentanyl citrate presents limitations regarding the detection of various fentanyl analogues, existing literature [40] suggests that spectral differences among these compounds are minimal, which may enhance the applicability of our findings. Moreover, while the presence of other medications in the donor population poses a risk of confounding results, our rigorous validation process indicates that the model effectively captures fentanyl-specific biochemical signatures. This proof-of-concept study provides the basis for future studies aimed at expanding the dataset to include a range of fentanyl analogues and improving model robustness for forensic applications. The exploration of this innovative methodology holds significant promise for enhancing the detection and understanding of fentanyl use in toxicology.

## Data Availability

The data that support the findings of this study are available on request from the corresponding author.
